# Long-Chain and Medium-Chain Fatty Acids in Energy Metabolism of Murine Kidney Mitochondria

**DOI:** 10.3390/ijms24010379

**Published:** 2022-12-26

**Authors:** Alexander V. Panov, Vladimir I. Mayorov, Anna E. Dikalova, Sergey I. Dikalov

**Affiliations:** 1 Department of Biomedical Sciences, Mercer University School of Medicine, Macon, GA 31201, USA; 2 Division of Clinical Pharmacology, Vanderbilt University Medical Center, Nashville, TN 37232, USA

**Keywords:** kidney, kidney mitochondria, respiration, oxidative phosphorylation, beta-oxidation, fatty acids, palmitoylcarnitine, octanoylcarnitine, succinate, succinate dehydrogenase, respirasome

## Abstract

Scientists have long established that fatty acids are the primary substrates for kidney mitochondria. However, to date we still do not know how long-chain and middle-chain fatty acids are oxidized at the mitochondrial level. Our previous research has shown that mitochondria from the heart, brain, and kidney oxidize palmitoylcarnitine at a high rate only in the presence of succinate, glutamate, or pyruvate. In this paper, we report properties of the isolated kidney mitochondria and how malate and succinate affect the oxidation of C16 and C8 acylcarnitines. The isolated kidney mitochondria contain very few endogenous substrates and require malate to oxidize pyruvate, glutamate, and C16 or C8 acylcarnitines. We discovered that with 10 µM of C16 or C8 acylcarnitines, low concentrations of malate (0.2 mM) or succinate (0.5 mM) enhance the States 4 and 3 respiratory rates several times. The highest respiration rates were observed with C16 or C8 acylcarnitines and 5 mM succinate mixtures. Results show that kidney mitochondria, unlike the heart and brain mitochondria, lack the intrinsic inhibition of succinate dehydrogenase. Additionally, results show that the oxidation of fatty acid by the small respirasome’s supercomplex generates a high level of CoQH2, and this makes SDH in the presence of succinate reverse the flow of electrons from CoQH2 to reduce fumarate to succinate. Finally, we report evidence that succinate dehydrogenase is a key mitochondrial enzyme that allows fast oxidation of fatty acids and turns the TCA cycle function from the catabolic to the anabolic and anaplerotic metabolic pathways.

## 1. Introduction

A kidney’s central structural and functional units are nephrons, which have the filtering part, glomerulus, and tubules where reabsorption occurs. Nephrons are located in the kidney’s cortex [1]. The kidney has a high density of mitochondria, yet despite elucidation of the delicate and complex molecular structures of the glomeruli and tubules [2], the metabolic functions of kidney mitochondria have mostly remained an enigma. Glomerular filtration of the blood plasma with the primary urine formation does not require ATP or other metabolic energy. The driving force for glomerular filtration is the transcapillary difference in hydrostatic and oncotic pressures [3]. In the tubular part of a nephron, almost all metabolites and solutes, such as glucose, amino acids, and sodium ions, reabsorb back into the blood, which requires an enormous amount of energy [4]. In addition to these excretory, ion, and osmolarity maintenance functions, kidneys also require energy to produce a large amount of glucose for maintaining glucose homeostasis, together with the liver during starvation [5].

The process of glucose reabsorption is tightly bound to the symport of sodium. Over 1.6 kg of salt is filtered, but only 3–20 g is excreted with urine, and significant energy is expended to conserve such a large fraction of water and salt in addition to glucose and other filtered solutes [6]. The glucose-Na^+^ symport is driven by active sodium extrusion by the basolateral sodium/potassium-ATPase, thus facilitating glucose uptake against an intracellular uphill gradient. Basolaterally, glucose exits the cell through facilitative glucose transporter 2 (GLUT2). More than 90% of glucose-Na^+^ symport occurs in the proximal tubule segments 1 and 2 (S1/2) [7]. Some researchers regard these two proximal tubule segments as the most ATP-demanding human body part [8]. Thus, kidney proximal tubule mitochondria must not only provide large amounts of ATP, but also at a very high rate. In addition, normal proximal tubules do not utilize glucose as a source of energy [9]. In recent years it has been established that abnormalities of fatty acid metabolism in the kidneys result in chronic and acute kidney injury [4,10,11,12,13].

Physiologists have long since established that in the kidneys, fatty acids serve as a predominant substrate for the production of ATP [12,13]. However, at the mitochondrial level, β-oxidation of long-chain fatty acids remains practically unstudied. This notion applies not only to the kidney mitochondria, but also to the brain and the heart mitochondria [14,15]. 

Earlier, we established that the isolated rat heart and brain synaptic mitochondria oxidize acylcarnitines of the long-chain fatty acids at high rates only in the presence of supporting substrates, which are mitochondrial metabolites, namely, succinate, glutamate, and pyruvate [16,17]. Recently, we have shown that the isolated kidney mitochondria from C57Bl/6N mice also efficiently oxidize palmitoylcarnitine in the presence of glutamate, pyruvate, or succinate [18]. However, the general metabolic properties of isolated kidney mitochondria remain understudied in comparison to the mitochondria from other high energy-consuming organs, such as the heart and brain [16,17]. Unlike the brain and heart mitochondria, which display a robust intrinsic inhibition of succinate dehydrogenase, the isolated kidney mitochondria oxidize succinate at high rates in all metabolic states [18]. With the kidney mitochondria, succinate was the most compelling substrate supporting the β-oxidation of palmitoylcarnitine (the most common C16 long-chain fatty acid) [18]. In this paper, we present new data on the general metabolic properties of kidney mitochondria from C57Bl/6J mice; we also analyze the effects of different concentrations of malate and succinate on oxidation of acylcarnitines of the long-chain (C16) and middle-chain (C8) fatty acids by kidney mitochondria. 

In this paper, we demonstrate that kidney mitochondria, similar to the synaptic mitochondria, have a very low content of endogenous substrates and thus strongly depend on the constant supply of external substrates. We conclude that succinate may serve for a limited time as an effective substrate for proximal tubule mitochondria in case of fatty acid metabolism failure, but the long-term high rates of ATP production occur when succinate and fatty acids are present together. We hypothesize the significance of succinate dehydrogenase and fatty acids oxidation in the hard-working organs, such as the kidney, heart, and brain, for the distribution of energy and redox equivalents by the respirasome’s supercomplexes. 

## 2. Results

### 2.1. Endogenous Substrates

Freshly isolated mitochondria from various organs may have different amounts of endogenous substrates in the mitochondrial matrix. Extreme examples are mitochondria from the liver, which can respire for as long as 15 min without added substrates, whereas isolated synaptic mitochondria, commonly referred to as the brain mitochondria, have zero oxygen consumption without substrate supplementation [17,19]. The contents of endogenous substrates in mitochondria from the heart and kidney are between these extremes. The isolated kidney mitochondria are closer to the brain mitochondria, despite respiration being not precisely zero but very low without added substrates. 

In experiments in vitro with the isolated mitochondria, even with the added substrates, the composition and the amount of endogenous mitochondrial metabolites may affect respiration. For example, in the heart mitochondria, the oxidation rates of palmitoylcarnitine and malate strongly depend on endogenous mitochondrial metabolites that support the oxidation of fatty acids. Thus, the respiration rates depend on whether an experiment is performed shortly after the isolation or later [20]. 

**Figure 1:**
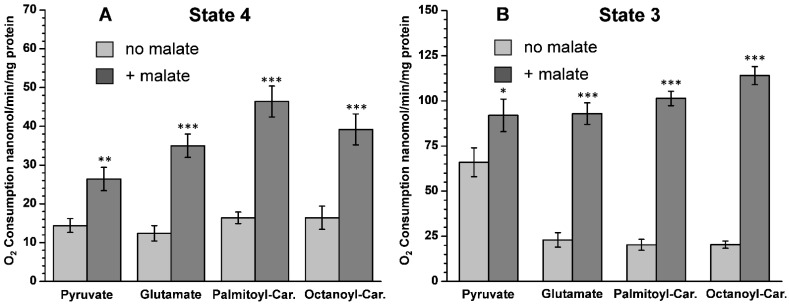
The addition of malate stimulates the oxidation of pyruvate, glutamate, C16, and C8 acylcarnitines by mouse kidney mitochondria. (**A**) The mitochondrial metabolic State 4 (resting respiration); (**B**) Metabolic State 3 (oxidative phosphorylation) initiated by adding 150 µM ADP. The incubation medium contained: 125 mM KCl, 10 mM MOPS, pH 7.2, 2 mM MgCl_2_, 2 mM KH_2_PO_4_, 10 mM NaCl, 1 mM EGTA, 0.7 mM CaCl_2,_ and if added, malate 2 mM. Substrates: Pyruvate 2.5 mM, glutamate 5.0 mM, L-palmitoylcarnitine 10 µM, L-octanoylcarnitine 10 µM. Substrates were added before the addition of 0.3 mg kidney mitochondria. The addition of 150 µM ADP initiated State 3 respiration. The light gray columns represent the rate of O_2_ consumption with a substrate alone; the dark gray columns represent a substrate + malate. Consider the difference in the Y scale values between Figure 1A,B. Statistics: *—*p* < 0.5; **—*p* < 0.05; ***—*p* < 0.001. The number of experiments (separate isolations of kidney mitochondria) for each substrate: without malate *n* = 5; with malate *n* = 8.

Isolated kidney mitochondria oxidize succinate well without other metabolites present [18]. However, when pyruvate, glutamate, palmitoylcarnitine, or octanoylcarnitine were present as a sole substrate, the rates of respiration in both State 4 (resting respiration) and State 3 (phosphorylating respiration) were very slow (Figure 1). One of the reasons for poor respiration in the mouse kidney mitochondria with these substrates could be the lack of malate, a source of oxaloacetate. During mitochondrial metabolism, pyruvate and fatty acids produce acetyl-CoA, which requires oxaloacetate to form citrate, and thus enter the tricarboxylic acid (TCA) cycle. Glutamate requires oxaloacetate for transamination to aspartate and α-ketoglutarate, which is further oxidized in the TCA cycle with the formation of succinate. Figure 1A,B shows that upon adding 2 mM malate, the respiratory rates with these substrates increased dramatically. It must be noted that malate is a very poor mitochondrial substrate when used as the sole substrate. Thus, the data presented in Figure 1 suggest that kidney mitochondria contain very few endogenous substrates and, similar to the brain synaptic mitochondria, depend on a constant supply of external substrates.

### 2.2. Intrinsic Inhibition of Succinate Dehydrogenase (SDH)

From the early days of mitochondriology, it was shown that isolated mitochondria from the heart and brain poorly oxidized succinate. Inhibition of succinate dehydrogenase (SDH) was not observed when the mitochondria were isolated in the presence of defatted bovine serum albumin (BSA) [21]. For these reasons, for decades the intrinsic inhibition of SDH was considered an artifact of the isolation procedure. However, we have established that the intrinsic inhibition of SDH is a crucial physiological mechanism for preventing excessive reactive oxygen species (ROS) production under diminished functional activity of the heart or brain [19,21].

**Figure 2:**
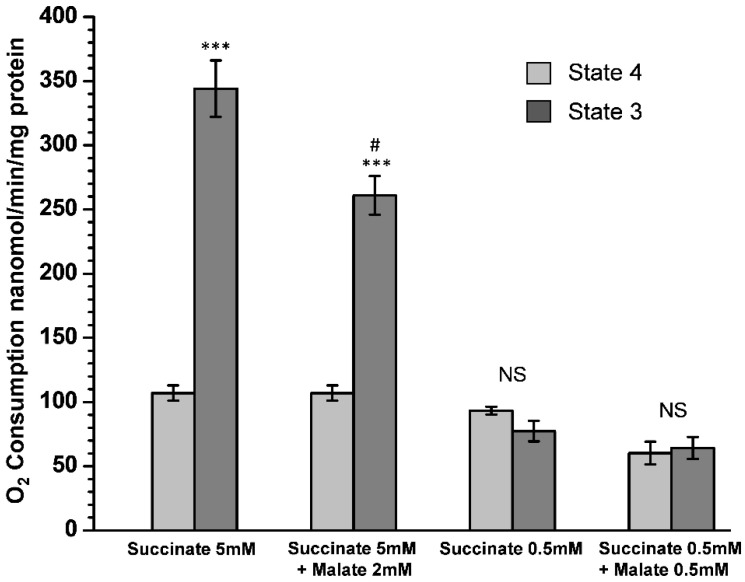
Kidney mitochondria do not possess the intrinsic inhibition of succinate dehydrogenase. The light gray columns represent O_2_ consumption rate in State 4, and the dark gray columns represent the respiration rates in State 3 initiated by adding 150 µM ADP. Incubation conditions as in Figure 1. Substrates: succinate and malate concentrations, as shown in the figure. Statistics: ***—*p* < 0.001; #—*p* < 0.001, a comparison with the State 3 succinate 5 mM. The number of experiments (separate isolations of kidney mitochondria) for each substrate: Succinate 5 mM, *n* = 7; succinate 5 mM + malate 2 mM, *n* = 5; succinate 0.5 mM, *n* = 6; succinate 0.5 mM + malate 0.5 mM, *n* = 4.

Figure 2 shows no inhibition of succinate oxidation in the resting (State 4) and phosphorylating (State 3) respiration in the freshly isolated kidney mitochondria. The simultaneous presence of malate with succinate does not affect resting respiration but does significantly diminish the oxidative phosphorylation rate (*p* < 0.01). Figure 2 also confirms that SDH has a low affinity to succinate, a known fact for the liver and heart mitochondria but unknown for the kidney mitochondria. Thus, SDH has a low affinity to succinate in the kidney mitochondria, which affects oxidative phosphorylation. Importantly, with 0.5 mM succinate, kidney mitochondria display a slightly lower State 4 respiration rate (*p* < 0.5), compared with 5 mM succinate. With 0.5 mM succinate there is no State 3 (oxidative phosphorylation) respiration.

### 2.3. Oxidation of L-Palmitoylcarnitine and L-Octanoylcarnitine

β-Oxidation of the long-chain and middle-chain fatty acids is the primary source of energy in the heart, liver, skeletal muscles, and kidney. Long-chain (C12–C20) saturated fatty acids undergo β-oxidation after being released during lipolysis of the white fat tissue triglycerides, whereas the middle-chain fatty acids are derived predominantly from triglycerides of some plant oils and milk [22,23,24,25]. Long-chain fatty acids with one or two double bonds are typically present in phospholipids and may serve as substrates for β-oxidation after release by the phospholipase A_1_, but their oxidation depends on the position of a double bond [26]. Polyunsaturated fatty acids, with three and more double bonds, are poorly oxidized by mitochondria and usually undergo oxidation in peroxisomes [26]. Acylcarnitines such as palmitoylcarnitine or octanoylcarnitine are commonly used as substrates for mitochondrial research as the most physiologically typical fatty acids [23].

**Figure 3:**
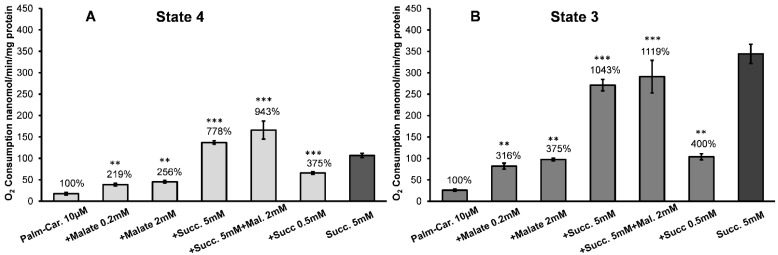
Effect of different concentrations of malate and succinate on oxidation of palmitoylcarnitine by the isolated kidney mitochondria. (**A**) Metabolic State 4; (**B**) Metabolic State 3. State 3 was initiated by adding 150 µM ADP. Incubation conditions as in Figure 1. The oxygen consumption rates by mitochondria oxidizing palmitoylcarnitine alone in the States 4 and 3 were taken as 100%. The actual respiration rates with 10 µM palmitoylcarnitine were: State 4, 18 ± 1.6, and State 3, 26 ± 1.8 nanomol O_2_/min/mg protein. Statistics: **—*p* < 0.05; ***—*p* < 0.001. The number of experiments (separate isolations of kidney mitochondria) for each substrate: palmitoylcarnitine, *n* = 7; palm.-carn. + malate 0.2 mM, *n* = 7; palm.-carn. + malate 2 mM, *n* = 8; palm.-carn. + succinate 5 mM, *n* = 8; palm.-carn. + succinate 0.5 mM, *n* = 8; succinate 5 mM, *n* = 7.

**Figure 4:**
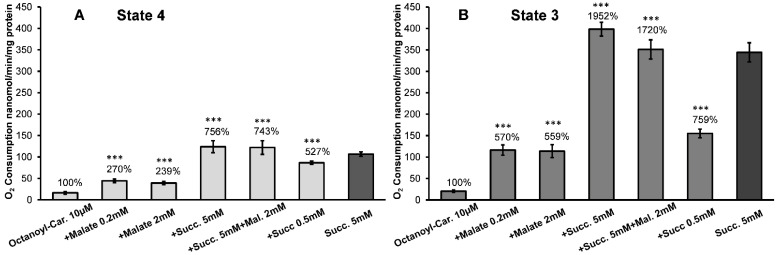
Effects of different concentrations of malate and succinate on oxidation of octanoyl-carnitine by the isolated kidney mitochondria. (**A**) Metabolic State 4; (**B**) Metabolic State 3. Incubation conditions as in Figure 2 and Figure 3. The oxygen consumption rates by mitochondria oxidizing octanoylcarnitine alone in the States 4 and 3 were taken as 100%. The actual respiration rates with 10 µM octanoylcarnitine were: State 4, 16 ± 2.6, and State 3, 20 ± 1.7 nanomol O_2_/min/mg protein. Statistics: ***—*p* < 0.001. The number of experiments (separate isolations of kidney mitochondria) for each substrate: octanoyl-carnitine, *n* = 6; octan.-carn. + malate 0.2 mM, *n* = 6; octan.-carn. + malate 2 mM, *n* = 6; octan.-carn. + succinate 5 mM, *n* = 6; octan.-carn. + succinate 0.5 mM, *n* = 6; succinate 5 mM, *n* = 7.

In the in vitro system with isolated mitochondria from the brain, heart, and kidney, the rates of oxidative phosphorylation with the long-chain acylcarnitines and malate as substrates are low, as shown in Figure 3 and Figure 4, see also [16,17,18,22]. The addition of an uncoupler results in the inhibition of respiration [16,17,18]. For these reasons, researchers rarely used long-chain fatty acids, such as palmitoylcarnitine, as substrates for mitochondrial energization.

Thus, there exists a discrepancy between experimental knowledge of the mechanisms of β-oxidation of fatty acids by the isolated mitochondria, and the fact that in vivo mitochondria in the heart and kidney utilize long-chain and middle-chain fatty acids as the primary source of energy [12,15,23]. We have suggested that succinate plays a crucial role in facilitating the β-oxidation of fatty acids, and the effectiveness of pyruvate and glutamate depends on their conversion to α-ketoglutarate and then to succinate [18]. In Figure 3, we present the results of experiments on stimulation of oxidation of 10 µM palmitoylcarnitine by the low and high concentrations of malate (0.2 mM and 2.0 mM) and succinate (0.5 mM and 5.0 mM) during resting respiration (State 4) and active oxidative phosphorylation (State 3). Figure 4 presents the results of similar experiments with octanoylcarnitine.

Resting respiration (State 4) is the respiration of the non-phosphorylating mitochondria. In experiments, we typically observe State 4 respiration before adding ADP and after the mitochondria phosphorylate. In the Percoll-purified mitochondria from the brain, kidney, and heart, both State 4 respiration rates are almost identical, indicating mitochondrial structural integrity and lack of contamination with damaged mitochondria. 

Figure 3 shows that adding a low concentration of malate (0.2 mM) to mitochondria oxidizing 10 µM palmitoylcarnitine resulted in a 2-fold activation of the State 4 oxygen consumption (Figure 3A) and a more than a 3-fold increase in oxidative phosphorylation (Figure 3B). A high malate level (2 mM) was only slightly more effective than 0.2 mM of malate. However, the stimulation of oxidative phosphorylation by malate is too small to satisfy the energy needs of the kidneys.

Figure 2 shows that succinate cannot support oxidative phosphorylation at a concentration of 0.5 mM. However, when mitochondria oxidized L-palmitoylcarnitine in the presence of 0.5 mM succinate, the State 4 oxygen consumption rates increased more than 3-fold (Figure 3A), and the rate of oxidative phosphorylation increased 4-fold (Figure 3B).

The addition of 5 mM succinate as a supporting substrate increased respiration of the kidney mitochondria 8-fold in State 4 (Figure 3A) and 10-fold in State 3 (Figure 3B). In the presence of succinate and 2 mM malate, the rates of O_2_ consumption increased even further.

Figure 4 presents similar to Figure 3 experiments with the kidney mitochondria oxidizing L-octanoylcarnitine. Compared with octanoylcarnitine alone, taken as 100%, adding 0.2 mM malate to the incubation medium increased almost 3-fold oxygen consumption rate in State 4 and more than 5-fold in State 3. As with the palmitoylcarnitine, a small (0.2 mM) malate concentration was sufficient to cause a maximal stimulation of C8 acylcarnitine oxidation in the metabolic States 4 and 3. Again, it is essential to note that with 0.5 mM succinate, the increases in respiratory rates were 5-fold in State 4 and more than 7-fold in oxidative phosphorylation. At a 0.5 mM concentration, succinate alone cannot support the State 3 respiration (see Figure 2). The addition of 5 mM succinate to octanoylcarnitine caused a 7-fold increase in oxygen consumption during resting respiration and almost a 20-fold increase in the rate of oxidative phosphorylation. In general, the state 3 respiration rates in the presence of 5 mM succinate were significantly higher with octanoylcarnitine (*p* < 0.01) than with palmitoylcarnitine (see Figure 3B and Figure 4B). Unlike experiments with palmitoylcarnitine (Figure 3B), the presence of succinate and malate caused a slight but significant (*p* < 0.1) inhibition of the State 3 oxidation of octanoylcarnitine (Figure 4B).

The data presented in Figure 3 and Figure 4 support our earlier conclusion that with fatty acids as substrates, high rates of oxidative phosphorylation can be achieved in the presence of an additional mitochondrial substrate, with succinate as the most effective stimulator of β-oxidation [16,17,18]. We have previously shown that in the brain and heart mitochondria, and more so in the kidney mitochondria, pyruvate was the least effective in stimulating the β-oxidation of palmitoylcarnitine [18]. These data align with the meager utilization of glucose by the kidney as a respiratory substrate. Under normal circumstances, only 2–3% of the ATP generated by the kidney is from glycolysis and only at the distal part of the tubules [27].

## 3. Discussion

### 3.1. Particular Qualities of Kidney Mitochondria and Their Significance for Kidney Functions

This work describes the metabolic properties of isolated mouse kidney mitochondria oxidizing long-chain and medium-chain fatty acids. One of the critical features of kidney mitochondria is that they are highly efficient in succinate utilization and do not possess the intrinsic inhibition of SDH observed in the brain and heart mitochondria (Figure 2 and [18]). Similar to brain mitochondria, kidney mitochondria contain almost no endogenous substrates. They, therefore, depend on the constant supply of substrates from the blood and glomerular filtrate, which is, before entering the proximal tubules, rich with various metabolites but poor in oxygen. The data presented indicate that malate is one of the critical metabolites necessary for the initiation of oxidation of pyruvate, glutamate, and fatty acids. We suggest that malate is a source of oxaloacetate, which is necessary for initiating the TCA cycle and glutamate transamination. Remarkably, malate is fully effective at a low concentration of 200 µM (Figure 3 and Figure 4). In this work and our previous publications [16,20], we show that malate is also a natural regulator of SDH activity and thus may control the β-oxidation of fatty acids and ROS production in the kidneys. The regulatory properties of malate are associated with the ability of SDH to oxidize, with the same affinity, both succinate and malate [25]. Oxidation of malate results in the formation of oxaloacetate, a potent inhibitor of SDH, directly on the active center. This property of SDH is determined genetically; therefore, there are wide phenotypic variations between species [20]. 

We suggest that the absence of intrinsic inhibition of SDH in the kidney mitochondria may be associated with the fact that, unlike the heart and brain, the kidneys work constantly and thus produce much less ROS as compared with the heart and brain, which have a high risk of oxidative stress at the periods of low functional activity. The other advantage of the high SDH activity for the kidney is that kidney mitochondria can utilize succinate for supporting glucose-Na^+^ symport in proximal tubules, which requires fast ATP production. Unfortunately, in vivo, the ample supply of succinate is limited. Only the β-oxidation of long-chain fatty acids can support the large and fast production of ATP for an extended period. Therefore, the primary function of succinate in kidney mitochondria is to enhance the rates of β-oxidation of fatty acids and redistribute energy released during the β-oxidation of fatty acids within the mitochondria and cells. 

Although the data presented in Figure 3 and Figure 4 seem to support the above hypothesis, one can argue that the high rates of respiration in the presence of C16 or C8 acylcarnitine and 5 mM succinate are associated with the oxidation of succinate but not the fatty acid. Several facts are evidence against this possibility. First, with the acylcarnitines and succinate as a sole substrate, adding an uncoupler causes inhibition of respiration. With the kidney, heart, or brain mitochondria oxidizing a mixture of fatty acylcarnitines and supporting substrates, the uncoupled respiration proceeds linearly at a high rate for a long time [16,17]. Secondly, 0.5 mM succinate, which alone cannot support oxidative phosphorylation, caused a several-fold stimulation of oxygen consumption with C16 or C8 acylcarnitines in the metabolic States 4 and 3 (Figure 3A,B and Figure 4A,B). Remarkably, with octanoylcarnitine, the stimulatory effect of 0.5 mM succinate significantly (*p* < 0.5) exceeds the effect of malate. Thirdly, the simultaneous oxidation of fatty acids and a supporting substrate stimulates a dramatic increase in oxygen consumption rate during resting (State 4) respiration. In the case of palmitoylcarnitine, the State 4 oxygen consumption significantly (*p* < 0.01) exceeds the rates observed during the oxidation of succinate alone (*p* < 0.01) (see Figure 2 and Figure 3). Earlier it was shown that the high rates of State 4 oxygen consumption during the oxidation of fatty acids or succinate were caused by increased production of ROS [17,19].

Recently, Anderson et al. [28] added radioactively labeled [U-^13^C]octanoic and [U-^13^C]decanoic acids to brain slices and observed the active metabolism of these middle-chain fatty acids [23]. Thus, Andersen et al. (2021) indirectly proved our earlier conclusion that fatty acids require not only the activation to acylcarnitines but also additional metabolites, which are present in the tissue slices, for the active β-oxidation by mitochondria [16,18,28]. Meanwhile, future studies with the simultaneous oxidation of fatty acids and supporting substrates using radioactively labeled substrates can demonstrate specific utilization of fatty acids in the presence of supporting substrates and thus resolve the arising uncertainty.

### 3.2. β-Oxidation of Fatty Acids and SDH Determine the Kidney’s Metabolic Phenotype and Increase the Extracellular Succinate Concentration

To understand the physiological significance of the mechanisms of energy production from the three primary energy sources, namely carbohydrates, fatty acids, and amino acids, we have to consider several related discoveries not fully appreciated until now. Let us briefly consider the following discoveries. In 2001, Schagger and Pfeiffer demonstrated that mitochondrial electron-transferring proteins are organized in three large supercomplexes forming a functional entity named a respirasome [29,30]. Two similar large supercomplexes contain complex I, a dimer of complex III, and two dimers of complex IV. These supercomplexes can directly oxidize only NADH generated in the mitochondria. The smaller supercomplex consists of one dimer of complex III and two dimers of complex IV, and it directly interacts with and oxidizes the membrane’s pool of reduced coenzyme Q (Co-QH_2_). The physiological significance of each component of the respirasome has not yet been discussed and understood.

A fundamental discovery from Brand and his team revealed that β-oxidation of long-chain fatty acids creates the highest level of reduction of the inner membrane’s pool of coenzyme Q. At high Co-QH_2_ content in the membrane, the mitochondrial succinate dehydrogenase, often called complex II, reverses transport of electrons from the ubiquinol (Co-QH_2_) and reduces fumarate to succinate. This specific reversed electron transport at SDH is inhibited explicitly by the antibiotic atpenin, but not by other inhibitors of SDH [31,32,33].

Further research from Wang et al. [34] proved that multifunctional fatty acid oxidation complexes within mitochondria are physically associated with the respirasome’s supercomplexes and promote metabolic channeling. Palmitoyl-CoA and octanoyl-CoA provide reducing equivalents NADH and Co-QH_2_ to the respirasome at the level of complexes I and III, and no accumulation of their intermediates was detected [34].

He et al. [35] reported that one of the G-protein-coupled receptors (GPCR), named GPR91, functions as a receptor for the citric acid cycle intermediate succinate. Later it was discovered that GPR91 is expressed in kidney, liver, heart, and retinal cells, leading to a wide array of physiological and pathological effects. Through GPR91, succinate is involved in functions such as regulating blood pressure, inhibiting lipolysis in the white adipose tissue, retinal vascularization, cardiac hypertrophy, and activating the hepatic stellate cells by ischemic hepatocytes reviewed in [36,37].

A final relevant discovery worth noting, Panov found that isolated mitochondria from the heart, brain, and kidney oxidize at high rates long-chain and middle-chain fatty acids only in the presence of additional mitochondrial metabolites: succinate, glutamate, or pyruvate [16,17,18].

Below, we present a hypothesis that incorporates the abovementioned discoveries. Of all the enzymes of the tricarboxylic acids cycle, only SDH, also known as the respiratory complex II, deeply embeds into the lipid core of the inner membrane; there may also be two SDH molecules for each complex I [38,39]. The active center of SDH opens into the matrix and contains FAD as a cofactor. Upon reduction of FAD during oxidation of succinate, three Fe-S clusters carry electrons one by one deep into the inner membrane’s lipid core, reducing the membrane’s pool of coenzyme Q to QH_2_. In the absence of β-oxidation of fatty acids, succinate oxidation catalyzed by SDH is irreversible because the minor supercomplexes instantly and irreversibly oxidize QH_2_.

**Figure 5:**
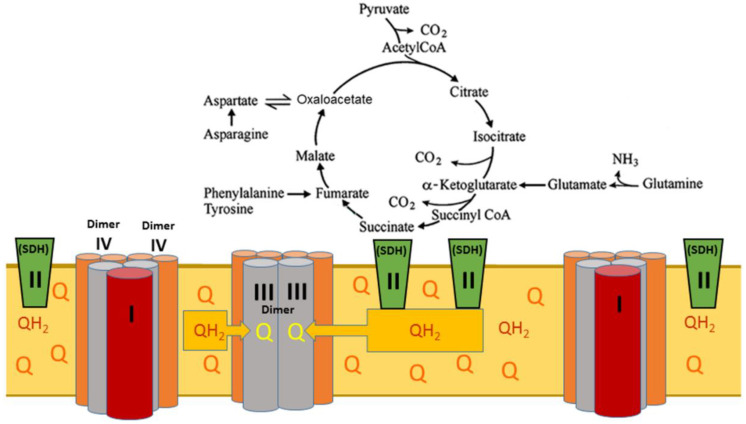
Succinate dehydrogenase (SDH), also known as respiratory complex II, is the only intramembrane enzyme of the TCA cycle. In the absence of fatty acid β-oxidation, SDH is the TCA cycle’s point of irreversibility, thus, enhancing its catabolic disposal of metabolites and producing ATP and heat at moderate rates, faster than the respiration supported by the NADH-dependent substrates. Under these conditions, the respiration rate is limited by the SDH activity as the primary source of CoQH_2_. Q—ubiquinone, the oxidized form of Coenzyme Q; QH_2_—ubiquinol, the reduced form of Coenzyme Q.

Figure 5 shows the interactions of the respirasome’s components with the enzymes of the TCA cycle in the absence of β-oxidation of fatty acids when mitochondria oxidize pyruvate, succinate, and amino acids undergoing catabolic transformations. First, it is important to note the known kinetic and metabolic differences between the large and the minor supercomplexes. In a large supercomplex containing complex I, the flow of electrons down the respiratory chain begins from the NADH dehydrogenase, the first enzyme of complex I, which is the rate-limiting reaction. Therefore, all respiratory components down the respiratory chain would be oxidized if NADH was the sole substrate [37]. However, in real life, mitochondria simultaneously oxidize multiple substrates. Many amino acids, glutamate, histidine, proline arginine, and others degrade to α-ketoglutarate and then succinate, thus, inputting more electrons into the respiratory chain. Glutamate and pyruvate cannot be regarded as strict complex I substrates because they undergo transamination in the brain, heart, and kidney, producing succinate and driving complex II respiration. Succinate oxidation provides a much faster oxidation rate and can drive reverse electron transport during resting respiration [19,20]. At high energization of mitochondria, the flow of electrons in complex I may reverse, reducing the sites which produce superoxide radicals [40,41]. Interestingly, with glutamate and malate as substrates, at least 58% of glutamate oxidation in kidney mitochondria is driven by complex II, supporting SDH’s critical role in kidney metabolism [42].

**Figure 6:**
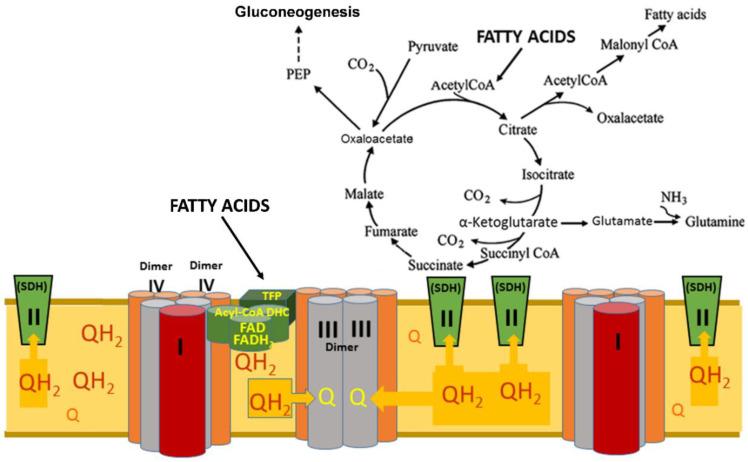
β-Oxidation of fatty acids generates a very high level of Co-QH_2_ in the inner membrane, which reverses the flow of electrons at the SDH and thus turns the TCA cycle from the catabolic to anabolic and anaplerotic metabolic pathways. Abbreviations: Acyl-CoA DHC—acyl-CoA dehydrogenase complex, which comprises three enzymes: acyl-CoA dehydrogenase, electron transfer flavoprotein (ETF), electron-transferring-flavoprotein dehydrogenase (ETFDH); PEP—phosphoenolpyruvate; TFP—trifunctional protein of the β-oxidation system; SDH—succinate dehydrogenase, also known as complex II; Q—ubiquinone, the oxidized form of Coenzyme Q; QH_2_—ubiquinol, the reduced form of Coenzyme Q.

According to Brand, during β-oxidation of LCFAs, mitochondria reduce the matrix pool of NAD^+^ to NADH + H^+^ and the membrane’s pool of ubiquinone to ubiquinol (CoQH_2_) [31,32,33]. Note that the enzymes involved in the β-oxidation of fatty acids are also arranged into two polyenzymatic complexes, which are physically associated with the respirasome [34]. The fundamental discovery made by Brand and his team was that in the mitochondria, β-oxidation of fatty acids generates the highest level of CoQ reduction. The electrons from the CoQH_2_ at the respiratory complex II (SDH) become reversed and reduce fumarate to succinate (Figure 6). Thus, in the mitochondrial matrix, succinate accumulates, and the TCA cycle switches from the catabolic metabolic pathway to anabolic and anaplerotic pathways [31,32,33]. In well-energized mitochondria, the excess electrons reduce components of complex I and thus accelerate the production of superoxide radicals [16,17]. Under these conditions, the SDH also produces ROS at a high rate [31,32,33].

Most mammalian cells possess the mitochondrial dicarboxylate carrier that mediates electroneutral export of succinate across the mitochondrial inner membrane [41]. Thus, upon accumulation of succinate in the mitochondrial, the cytosolic and nucleus pools of succinate become equilibrated. GPR91, sometimes referred to as succinate receptor 1 (SUCNR1), faces the extracellular environment and responds to succinate with a half-maximum effective concentration of 28–56 μM. The highest succinate concentration reported for extracellular fluids was 200 µM [41]. GPR91 is expressed in many tissues, including blood cells, adipose tissue, the liver, the retina, and the kidney [37]. GPR91 and its succinate ligand are novel detectors of local stress, including ischemia, hypoxia, toxicity, and hyperglycemia. Local levels of succinate in the kidney also activate the renin-angiotensin system. GPR91 may play a vital role in developing hypertension and the complications of diabetes mellitus, metabolic disease, and liver damage [37].

Thus, in the organs consuming a large amount of ATP, only β-oxidation of fatty acids can maintain the high rates of oxidative phosphorylation for a long time and simultaneously maintain the anabolic and anaplerotic metabolic pathways for maintaining the structure of cells and their numerous functions. In the kidneys, this notion is particularly relevant to podocytes. During β-oxidation of fatty acids, the minor supercomplexes are the main driving force of energization because the dimers of complexes III directly oxidize CoQH_2_ at a very high rate. Notably, the temperature close to the respirasome is around 50 °C [43]; thus, the lipid phase of the inner membrane is liquid. Therefore, the rates of diffusion of CoQ and CoQH_2_ are very high.

At high mitochondrial energization, the large supercomplexes of the respirasome maintain anaplerotic reactions, such as aerobic gluconeogenesis [18] and anabolic processes in the cytoplasm, which require NADPH. Energy-dependent mitochondrial nicotinamide nucleotide transhydrogenase, is responsible for maintaining the cells’ high NADPH/NADP^+^ ratio [44,45]. The highest expression of this enzyme was observed in the heart and kidney [44], which utilize β-oxidation of FAs as their primary energy source.

## 4. Materials and Methods

### 4.1. Animals

All experimental procedures with mice C57Bl/6J were approved by Vanderbilt and Mercer Institutional Animal Care and Use Committees.

### 4.2. Isolation of Mitochondria

To isolate kidney mitochondria, two kidneys from a C57Bl/6J mouse were cleaned from fat, cut into small pieces, and subjected to disintegration with polytron with two pulses for 2 s. Further procedures were similar to the isolation of the heart or brain mitochondria [46,47]. For the kidney mitochondria, we have found that with the final centrifugation at 8000× *g*, the resulting mitochondria were of the same quality as mitochondria sedimented at 10,000× *g*, followed by purification with the discontinuous Percoll gradient. This paper presents data for the kidney mitochondria isolated without purification with Percoll.

The isolation medium contained 75 mM mannitol, 150 mM sucrose, 20 mM MOPS at pH 7.2, and 1 mM EGTA. The final suspensions of mitochondria were prepared using the incubation medium described below. Mitochondrial protein was determined with the Pierce Coomassie protein assay reagent kit.

### 4.3. Measurements of Mitochondrial Respiration

The respiration rates of kidney mitochondria were measured using Fluorescence Lifetime Micro Oxygen Monitoring System (Instech Laboratories, Inc., Plymouth Meeting, PA, USA). The incubation medium contained: 125 mM KCl, 10 mM MOPS at pH 7.2, 2 mM MgCl_2_, 2 mM KH_2_PO_4_, 10 mM NaCl, 1 mM EGTA, and 0.7 mM CaCl_2_. At a Ca^2+^/EGTA ratio of 0.7, the free [Ca^2+^] is close to 1 μM as determined using Fura-2. The substrate concentrations were: 0.5 mM or 5 mM succinate without rotenone, 0.2 mM or 2.0 mM malate, L-palmitoylcarnitine, and octanoylcarnitine were added in 5 µL aliquots giving a concentration of 0.01 mM. The stock solutions of C16 and C8 L-acylcarnitines were prepared in 50% ethanol. A total of 5 µL of the stock solution was added to the incubation chamber to obtain the final concentration of L-acylcarnitine of 10 µM. Control experiments showed no effect of ethanol on mitochondrial respiration. Substrates and substrate mixtures were added before the addition of 0.3 mg mitochondria. After 2 min of registration of the resting respiration (initial metabolic state 4), 150 µM of ADP was added to the incubation chamber (volume 1 mL). We did not use uncouplers because the electrode’s fluorescent sensor loses its sensitivity after washing with ethanol-containing solution. The data are presented as mean ± standard error of 5–9 separate experiments for each substrate and substrate mixture in 11 different isolations.

### 4.4. Chemicals

All chemicals were of the highest quality and purchased from Sigma-Aldrich Co., St. Louis, MO, USA. Glass bidistilled water was used to prepare the isolation and incubation media.

### 4.5. Statistics

Data are expressed as mean ± SEM. To compare the effect of substrates or ADP on mitochondrial respiration, two-way analysis of variance (ANOVA) was used followed with the Bonferroni post hoc test. For data involve more than two groups, one-way ANOVA followed with the Bonferroni post hoc test was used using GraphPad Prizm 7. *p* levels < 0.05 were considered significant.

## 5. Conclusions

Kidneys work constantly and require a large quantity of ATP. For this reason, kidney mitochondria possess properties distinct from the mitochondria of other hard-working organs. Mitochondria in segments 1 and 2 of the kidney proximal tubules must provide a vast amount of ATP in a short time since more than 90% of glucose-Na symport occurs in this part of the proximal tubules. The structure of the mitochondrial respirasome suggests that constant and vast production of ATP mitochondria can be provided only during the β-oxidation of fatty acids. Kidney mitochondria, similar to the heart and brain mitochondria, oxidize long-chain and middle-chain fatty acids only in the presence of other mitochondrial substrates. Succinate is the most effective substrate in supporting fatty acid β-oxidation in the kidney mitochondria. We suggest that succinate dehydrogenase is a key mitochondrial enzyme that allows fast oxidation of fatty acids and turns the TCA cycle function from the catabolic to the anabolic and anaplerotic metabolic pathways. Thus, during the β-oxidation of fatty acids, mitochondria provide enough energy not only to satisfy numerous kidney functions but for the maintenance of the structural integrity of the kidney.

## Abbreviations

ADP—adenosine diphosphate; ATP—adenosinetriphosphate;, ATPase—adenosinetriphosphatase; BSA—bovine serum albumin; CoQ—oxidized coenzyme Q, ubiquinone CoQH_2_—reduced coenzyme Q, ubiquinol; EGTA—ethyleneglycoltetraacetic acid; ETF—electron transfer flavoprotein; ETFDH—electron-transferring-flavoprotein dehydrogenase; GLUT2—glucose transporter 2; GPR91—G-protein-coupled succinate receptor; MOPS—3-(N-Morpholino)propanesulfonic acid; NAD^+^—nicotinamide adenine nucleotide, oxidized; NADH nicotinamide adenine nucleotide, reduced; NADPH—nicotinamide adenine nucleotide phosphate, reduced; TCA cycle—tricarboxylic acids cycle; SDH—succinate dehydrogenase; ROS—reactive oxygen species; PEP—phosphoenolpyruvate; TFP—trifunctional protein of the β-oxidation system.
